# Supplementary material for: Class-I myosin responds to changes in membrane tension during clathrin-mediated endocytosis in human induced pluripotent stem cells
Source: Proc Natl Acad Sci U S A. 2026 Feb 24;123(9):e2532817123. doi: 10.1073/pnas.2532817123 (PMC12956820; doi:10.1073/pnas.2532817123)
Supplement: Supplementary file 1 — Appendix 01 (PDF) [file pnas.2532817123.sapp.pdf]

Supplemental Information

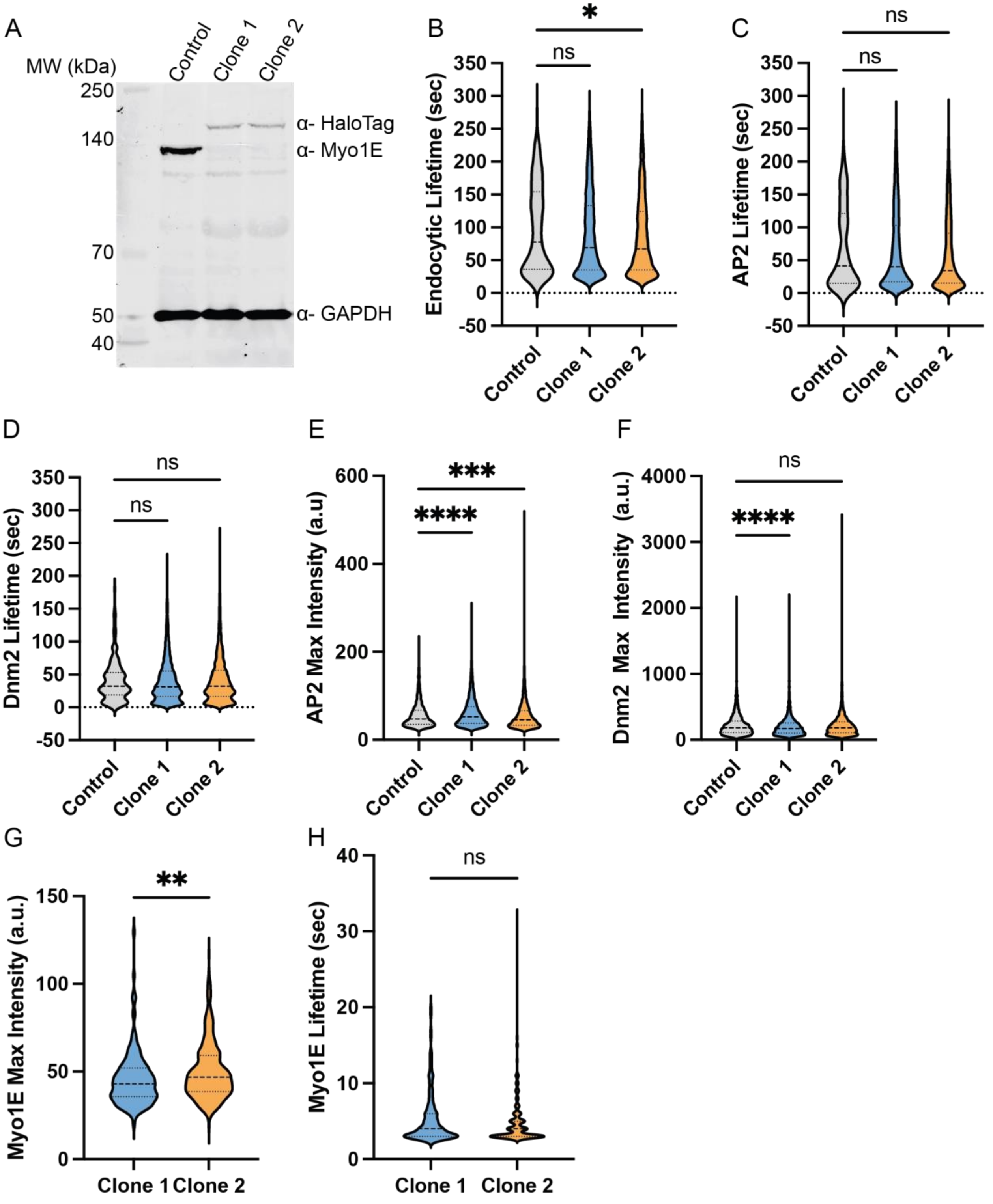

Figure S1. (A) Western blot of Myo1E-HaloTag knock-in. AP2-tagRFP-T; DNM2-tagGFP2 hiPSC line is the parent control cell line. Myo1E-HaloTag is 160 kDa, Myo1E endogenous is 127 kDa and GAPDH is 36 kDa. (B-G) Volin plots

comparing control AP2-tagRFP-T; DNM2-tagGFP2 hiPSCs with two AP2-tagRFP-T; DNM2-tagGFP2; MYO1E-JF635 clones generated from clonal expansion after genome-editing MYO1E with a HaloTag. Clone 2 was used for these studies as it had a more similar Dnm2-GFP maximum intensity to the control compared to Clone 1.

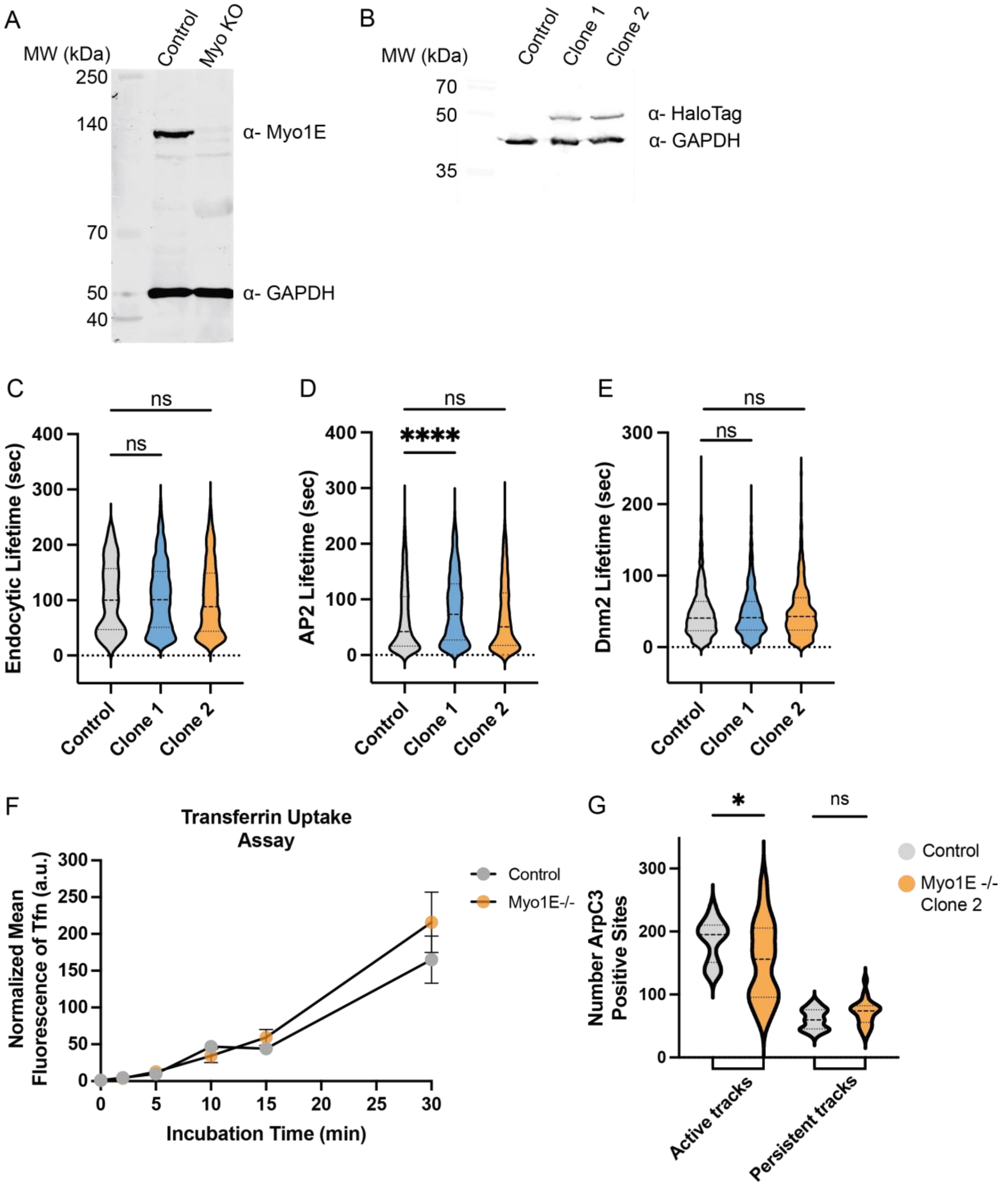

Figure S2. (A) Western blot of AP2-tagRFP-T; DNM2-tagGFP2 control hiPSCs and homozygous Myo1E knockout hiPSCs in an AP2-tagRFP-T; DNM2-tagGFP2 background. Myo1E endogenous is 127 kDa and GAPDH is 36 kDa. (B)

Western blot of AP2-tagRFP-T; DNM2-tagGFP2 control hiPSCs and ArpC3-HaloTag knock-in in Myo1E knockout hiPSCs. ArpC3-HaloTag is 54 kDa and GAPDH is 36 kDa. (C-E) Volin plots comparing control AP2-tagRFP-T; DNM2-tagGFP2; ArpC3-HaloTag hiPSCs with two AP2-tagRFP-T; DNM2-tagGFP2; ArpC3-HaloTag; MYO1E knockout clones generated from clonal expansion after knocking out MYO1E and tagging ArpC3 with a HaloTag. Clone 2 was used for these studies as it had more similar AP2-RFP lifetimes compared to the control. (F) Time-course transferrin-647 uptake assay of control cells compared to knockout cells. (G) Violin plot showing the number of endocytic sites in control cells and Myo1E-/- cells for both active sites and persistent sites.

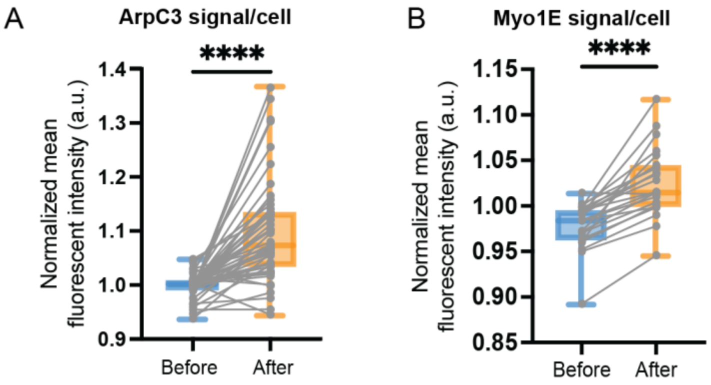

Figure S3. (A) Box and whisker plot of the mean fluorescent intensity of ArpC3 for the time before and the time after aspiration. The grey dots represent the individual mean values for each cell connected with the mean values after aspiration for the same cell (P**** < 0.0001, for the Wilcoxon test). (B) Box and whisker plot of the mean fluorescent intensity of Myo1E for the time before and the time after aspiration (P**** < .0001, for the Wilcoxon test).

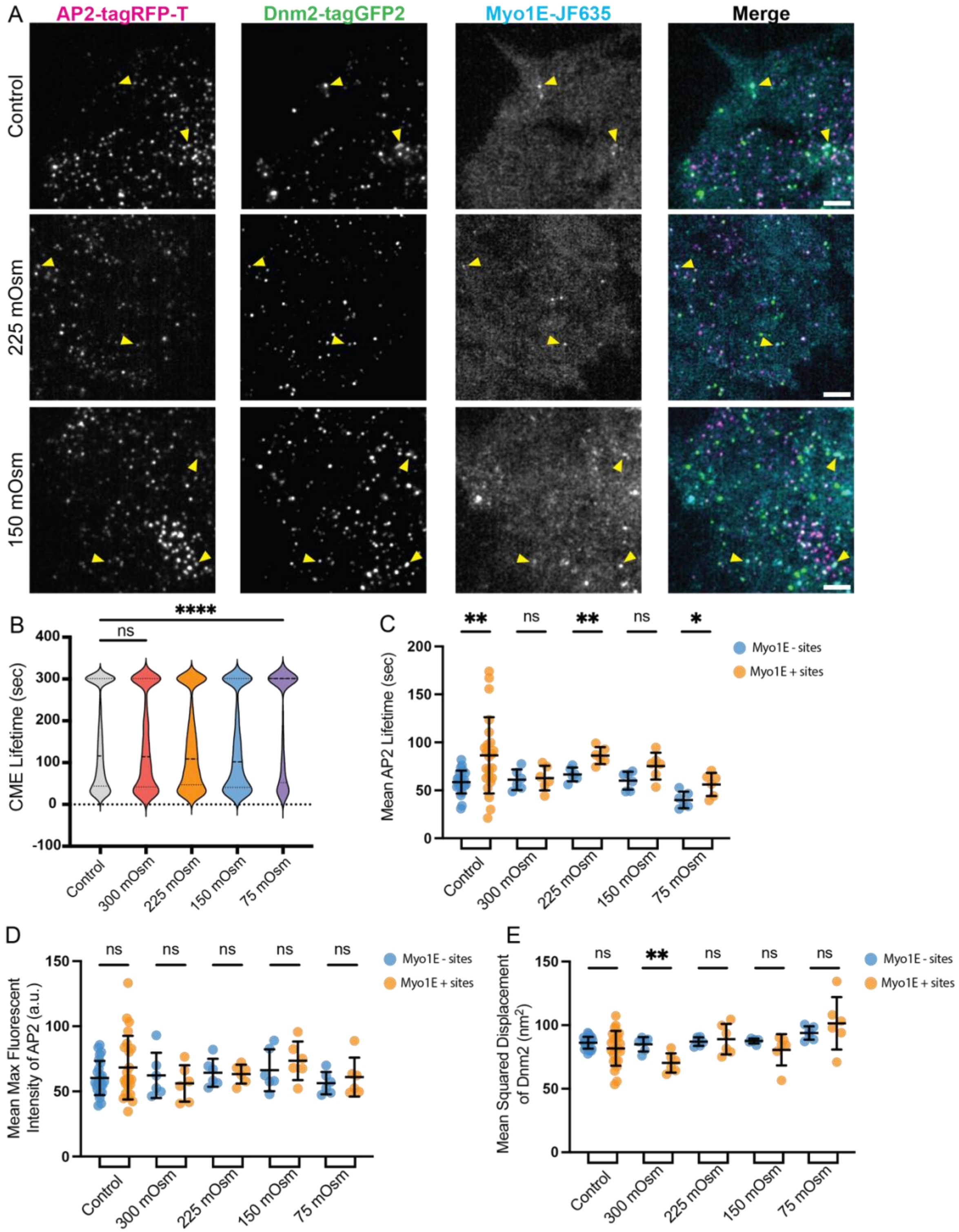

Figure S4. (A) TIRF image of ADM cells after the addition of hypotonic media to reach a final concentration of 225 and 150 mOsm. Scale bar = 5 µm. (B) Violin plot of CME lifetimes of all AP2-RFP;DNM2-GFP sites under varying osmotic shock conditions (P****< 0.0001, for Kruskal-Wallis). (C-D) Scatterplots characterizing AP2-RFP lifetime, mean fluorescent intensity, and mean displacement between endocytic sites that are Myo1E negative and Myo1E positive. (P**** < 0.0001, for Mann-Whitney). Error bars show SD.

Movie 1. TIRF movie of hiPSCs endogenously expressing AP2-tagRFP-T (magenta), DNM2-tagGFP2 (green), and Myo1E-JF635 (cyan). Related to Fig. 1 (A-D). Frame rate is 1 frame per second (fps) and played back at 10 fps. Scale bar = 5 μm.

Movie 2. TIRF movie of hiPSCs endogenously expressing AP2-tagRFP-T (magenta), DNM2-tagGFP2 (green), and ArpC3-JF635 (cyan). Related to Fig. 2 (A-E). Cells are in a wildtype background. Frame rate is 1 frame per second (fps) and played back at 10 fps. Scale bar = 5 μm.

Movie 3. TIRF movie of hiPSCs endogenously expressing AP2-tagRFP-T (magenta), DNM2-tagGFP2 (green), and ArpC3-JF635 (cyan). Related to Fig. 2 (A-E). Cells are in a Myo1E-/- background. Frame rate is 1 frame per second (fps) and played back at 10 fps. Scale bar = 5 μm.

Movie 4. TIRF movie of hiPSCs endogenously expressing AP2-tagRFP-T (magenta), DNM2-tagGFP2 (green), and ArpC3-JF635 (cyan). Related to Fig. 3 (A-C). Cells are in a wildtype background. Frame rate is 1 frame per second (fps) and played back at 10 fps. Scale bar = 5 μm.

Movie 5. TIRF movie of hiPSCs endogenously expressing AP2-tagRFP-T (magenta), DNM2-tagGFP2 (green), and Myo1E-JF635 (cyan). Related to Fig. 3 (D-F). Cells are in a wildtype background. Frame rate is 1 frame per second (fps) and played back at 10 fps. Scale bar = 5 μm.

Movie 6. TIRF movie of hiPSCs endogenously expressing AP2-tagRFP-T (magenta), DNM2-tagGFP2 (green), and Myo1E-JF635 (cyan) in 300 mOsm. Related to Fig. 4 (A-G). Cells are in a wildtype background. Frame rate is 1 frame per second (fps) and played back at 10 fps. Scale bar = 5 μm.

Movie 7. TIRF movie of hiPSCs endogenously expressing AP2-tagRFP-T (magenta), DNM2-tagGFP2 (green), and Myo1E-JF635 (cyan) in 75 mOsm. Related to Fig. 4 (A-G). Cells are in a wildtype background. Frame rate is 1 frame per second (fps) and played back at 10 fps. Scale bar = 5 μm.

Movie 8. TIRF movie of hiPSCs endogenously expressing AP2-tagRFP-T (magenta), DNM2-tagGFP2 (green), and ArpC3-JF635 (cyan) in 300 mOsm. Related to Fig. 5 (A, C-G). Cells are in a wildtype background. Frame rate is 1 frame per second (fps) and played back at 10 fps. Scale bar = 5 μm.

Movie 9. TIRF movie of hiPSCs endogenously expressing AP2-tagRFP-T (magenta), DNM2-tagGFP2 (green), and ArpC3-JF635 (cyan) in 75 mOsm. Related to Fig. 5(A, C-G). Cells are in a wildtype background. Frame rate is 1 frame per second (fps) and played back at 10 fps. Scale bar = 5 μm.

Movie 10. TIRF movie of hiPSCs endogenously expressing AP2-tagRFP-T (magenta), DNM2-tagGFP2 (green), and ArpC3-JF635 (cyan) in 300 mOsm. Related to Fig. 5 (B, C-G). Cells are in a Myo1E-/- background. Frame rate is 1 frame per second (fps) and played back at 10 fps. Scale bar = 5 μm.

Movie 11. TIRF movie of hiPSCs endogenously expressing AP2-tagRFP-T (magenta), DNM2-tagGFP2 (green), and ArpC3-JF635 (cyan) in 75 mOsm. Related to Fig. 5 (B, C-G). Cells are in a Myo1E-/- background. Frame rate is 1 frame per second (fps) and played back at 10 fps. Scale bar = 5 μm.
